# Formulation Development of Fast Dissolving Microneedles Loaded with Cubosomes of Febuxostat: In Vitro and In Vivo Evaluation

**DOI:** 10.3390/pharmaceutics15010224

**Published:** 2023-01-09

**Authors:** Brijesh Patel, Hetal Thakkar

**Affiliations:** Centre for Post-Graduate Studies and Research in Pharmaceutical Sciences, Shri G.H. Patel Pharmacy Building, Faculty of Pharmacy, The Maharaja Sayajirao University of Baroda, Vadodara 390002, Gujarat, India

**Keywords:** gout, Febuxostat, cubosomes, Microneedles, xanthine oxidase inhibitors

## Abstract

Febuxostat is a widely prescribed drug for the treatment of gout, which is a highly prevalent disease worldwide and is a major cause of disability in mankind. Febuxostat suffers from several limitations such as gastrointestinal disturbances and low oral bioavailability. Thus, to improve patient compliance and bioavailability, transdermal drug delivery systems of Febuxostat were developed for obtaining enhanced permeation. Cubosomes of Febuxostat were prepared using a bottom-up approach and loaded into a microneedle using a micromolding technique to achieve better permeation through the skin. Optimization of the process and formulation parameters were achieved using our design of experiments. The optimized cubosomes of Febuxostat were characterized for various parameters such as % entrapment efficiency, vesicle size, Polydispersity index, Transmission electron microscopy, in vitro drug release, Small angle X-ray scattering, etc. After loading it in the microneedle it was characterized for dissolution time, axial fracture force, scanning electron microscopy, in vitro drug release, pore closure kinetics, etc. It was also evaluated for various ex vivo characterizations such as in vitro cell viability, ex vivo permeation, ex vivo fluorescence microscopy and histopathology which indicates its safety and better permeation. In vivo pharmacokinetic studies proved enhanced bioavailability compared with the marketed formulation. Pharmacodynamic study indicated its effectiveness in a disease-induced rat model. The developed formulations were then subjected to the stability study, which proved its stability.

## 1. Introduction

Gout is a systemic disease that results from the deposition of monosodium urate crystals (MSU) in the tissues. Increased serum uric acid (SUA) above a specific threshold is a main reason for the formation of uric acid crystals. MSU crystals can deposit in all the tissues, mainly present in and around the joints forming tophi [1,2]. According to the American College of Rheumatology, medication therapy of gout involves the use of analgesics, NSAIDs, corticosteroids, colchicine, xanthine oxidase inhibitors and uricosurics [3,4,5].

A xanthine oxidase inhibitor (e.g., Allopurinol and Febuxostat) is a substance that inhibits the activity of xanthine oxidase, an enzyme involved in purine metabolism. In humans, inhibition of xanthine oxidase reduces the production of uric acid, and thus they are indicated for the treatment of hyperuricemia and related medical conditions including gout [1,5]. Febuxostat (FBX) is a novel, potent, non-purine selective xanthine oxidase inhibitor and has been reported to be more effective in lowering and maintaining serum urate levels than allopurinol. It is potent and more selective than allopurinol, and can be used safely in patients with hypersensitivity reactions towards allopurinol. Febuxostat is available as a tablet dosage form in the market for once a day administration in 40, 80 and 120 mg strengths. The tablets of Febuxostat are marketed under the names of Fabulas, Feboxa, Febuget, Febucip etc. The oral bioavailability of Febuxostat is 38% and is affected by the presence of food. The oral bioavailability of FBX is hampered by its low (<15 μg/mL) aqueous solubility and extensive enzymatic degradation in the intestine and liver. Furthermore, FBX’s peak plasma concentration (C_max_) is reduced by 38–49% in the presence of food [6]. FBX has its shortcomings, for example, poor bioavailability, food dependent absorption, gut-wall metabolism and gastrointestinal disturbances (nausea, diarrhea, stomach pain, ulcers, vomiting) [7]. The delivery of the drug through transdermal delivery will avoid all these problems associated with the present marketed formulation.

The transdermal route of drug delivery was selected to overcome the various limitations associated with FBX as cited above. However, the use of this route of drug delivery is limited by the presence of the outermost nonviable layer of stratum corneum [8,9,10]. Therefore, to overcome this limitation several mechanical, chemical approaches or novel drug delivery systems have been reported [11,12]. Nisomal gel [13], ethosomes [14], nanoemulsion [15] and self-nanoemulsion-loaded transdermal film [7] have been reported for the transdermal delivery of Febuxostat.

In the present investigation, a novel drug delivery system (Cubosomes) of Febuxostat was developed and characterized due to its efficiency of deeper penetration. Moreover, cubosomes also have advantages such as the ease of preparation, high drug loading and entrapment, low cost of raw materials, better skin penetration, and the fact it is bioadhesive and biocompatible etc. Cubosomes are the colloidal dispersion (size ranging from 100 to 300 nm) made by dispersing the bicontinuous cubic liquid crystalline structures in an aqueous medium that have surface active agents [16,17]. Cubosomes have the ability to encapsulate a variety of drug molecules falling into the hydrophilic, lipophilic and amphiphilic classes [18]. However, the use of cubosomes or any other nanocarrier systems does not ensure the complete permeation of therapeutic molecules across the skin which will decrease the bioavailability of drug molecules as reported in many literatures [19,20,21]. To improve the permeation across the skin, a combination of two or more approaches of the enhancement of transdermal permeation was suggested in many of the literatures, among which the combination of a Microneedles (MNs) patch with nanocarriers is the most prominent way to improve permeation across the skin [19,20,22]. Zhang P. et al., reported the use of MNs combined with chitosan nanoparticles to improve the permeation of insulin across the skin. They obtained a 4.2 fold increase in the permeation of insulin across the skin compared with the chitosan nanoparticles [19].

MN patches are generally made up of small micron sized needles attached to the patch. The application of MN proves to be advantageous as it facilitates the permeation across the toughest barrier, the stratum corneum, without causing any kind of pain [23]. Various materials can be used in the fabrication of (MN) and they can be biodegradable or non-biodegradable [24]. Biodegradable MNs are prepared using materials such as amylopectin, polyvinyl alcohol, poly vinyl pyrrolidone, poly lactic acid, PLGA, etc., [23]. They are preferred over the non-biodegradable ones as the accidental breakage of them later during insertion may lead to complications such as sepsis due to the broken part of the MN [10,23]. Thus, for the present project, MNs were fabricated using hydrophilic biodegradable polymers [23,25,26,27,28].

The objective of the present study was to develop, optimize and compare cubosomal formulation of FBX and an MN patch loaded with cubosomes of FBX for the enhancement of bioavailability by circumventing the first pass metabolism of FBX and to avoid gastrointestinal disturbances.

## 2. Materials and Method

Febuxostat and Glyceryl Monooleate (GMO) were obtained as a gift sample from Ami drugs and specialty chemicals Pvt. Ltd., India and Mohini Organics, Mumbai, India, respectively. DermaStamp DTR-150 and YYR-150 (35 and 80 titanium MNs, respectively) were procured from Guangzhou Junguan Beauty Co. Ltd., Guangzhou, China. Poly-dimethylsiloxane (Sylgard^®^ 184) was procured from Dow corning, Midland, MI, USA. Polyvinyl alcohol (PVA)-6000 and Lactose were purchased from Acros Organic, New Jersey, USA and Hi-media, Mumbai, India, respectively. Acetonitrile (HPLC grade), and methanol (HPLC grade) were purchased from Rankem Fine Ltd., Mumbai, India. Formic acid (AR grade), Disodium hydrogen phosphate, potassium dihydrogen phosphate, sodium chloride, sodium hydroxide were obtained from Spectrochem Labs Ltd., Mumbai, India. Thiazolyl Blue Tetrazolium Bromide (MTT) was bought from Sigma-Aldrich, USA. Antibiotic antimycotic solution 100×, Gamma irradiated fetal bovine serum and DMEM (Dulbecco’s Modified Eagle Medium) were purchased form Hi-Media, Mumbai, India. Potassium oxonate was obtained from TCI, Chennai, India. Preparation of double distilled water was performed in the laboratory, filtered with 0.2 μ membrane filter (store airtight container) and utilized within a maximum of 7 days.

### 2.1. Preparation of FBX Cubosomes

Bottom-up approach was utilized for the preparation of Cubosomes of FBX. A QbD approach was employed for the optimization of cubosomes of FBX using particle size and entrapment efficiency as Critical Quality Attributes (CQAs). In this process, concentration of lipid and PVA was selected as Critical Material Attributes (CMAs). Optimized composition for FBX cubosomes was mentioned in Table 1. For the preparation of cubosomes of FBX two solutions were prepared: (A) organic phase and (B) aqueous phase. For the preparation of organic phase (A), X % *w*/*v* of Glyceryl monoolein (GMO) was taken in a 10 mL glass beaker and dissolved in 4 mL of ethanol, then 40 mg of FBX was added to it. For aqueous phase (B), Y % *w*/*v* of PVA was dissolved in 20 mL of water. Both solutions were initially kept at a temperature of 60 °C and were continuously stirred for 5–10 min. Afterward, organic phase was added to aqueous phase in a drop-wise manner with continuous stirring and the addition of organic phase was maintained at a rate of 1 mL/min and 1000 rpm on magnetic stirrer. The resulting medium was cooled down to room temperature and then continuously stirred for 30 min using a magnetic stirrer. This medium was then introduced to the rotary evaporator at a temperature of 50 °C under vacuum for removing ethanol from the dispersion and the volume of the prepared batch was reduced to 10 mL. The resulting Cubosomal dispersion was exposed to centrifugation with process parameters, i.e., for a period of 10 min at 5000 rpm and the temperature was set as 25 °C for facilitating the sedimentation of free drug. Care was taken while separating the supernatant of cubosomal dispersion so as to not disturb the free drug pellet which is deposited at the bottom of the centrifuge tube. Finally, the resulting separated cubosomal dispersion was stored for utilization in future tests in glass vials at room temperature [27,29].

### 2.2. Preparation of MN Patch

A QbD approach was employed for the optimization of cubosomal FBX MN using axial fracture force and dissolution time as CQAs and concentration of PVA and lactose as CMAs. Optimized composition for cubosomal FBX MN was mentioned in Table 1. For the preparation of MN matrix, polyvinyl alcohol (PVA) was used as a polymer at concentration of X % *w/v* and Lactose was used as a filler at a concentration of Y % *w/v*. Hot plate magnetic stirrer was employed for solubilizing optimum amounts of PVA and lactose in 10 mL of FBX cubosomal dispersion (3.4 mg/mL FBX). This mixture was gently stirred at a temperature of 50 °C on the hot plate magnetic stirrer. Further, this mixture was cooled and was brought back to ambient room temperature. The resulting 1 mL of PVA/Lactose blend solutions which contain approximately 3.4 mg of drug were shifted into PDMS micromolds which have capacity of 2 mL. In order to fill the cavities of MN of the micromolds, it was centrifuged at 3000 RPM at 25 °C for 10 min. Further, to ensure that the MN structure was properly hardened, micromolds were placed in vacuum desiccators for a period of 24 h to evaporate the water. Then, 1567 high adhesion double coated medical tape (3M™, St. Paul, Minnesota, USA) which had backing film on the opposite adhesive side was employed to extract the MN arrays from micromolds. MN patches prepared from the aforesaid procedure were kept in airtight containers and calcium oxide along with silica gel was employed as desiccants [30,31].

### 2.3. Characterization of Cubosomes

#### 2.3.1. Vesicle Size Analysis

Ref. [32] The dispersions of FBX-loaded cubosomes were diluted up to 10 times with pre-filtered distilled water. Further, the dispersions were taken into disposable sizing cuvette and the vesicle size and poly-dispersity index (PDI) were analyzed with the help of Nano-ZS zetasizer which calculates vesicle size and PDI based on dynamic light scattering (DLS). For the calculation of mean diameter of cubosomes, the instrument examines angular scattering of a laser beam during its passage through the dispersed cubosomal sample and use the Mie theory of light scattering.

#### 2.3.2. Zeta Potential Measurement

Ref. [32] Nano-ZS zetasizer by Malvern Instruments Ltd., Bristol, UK, was used for the analysis of zeta potential of FBX-loaded cubosomes. For this, the dispersion of FBX-loaded cubosomes was taken and was diluted up to 10 times and the dilution was performed using pre-filtered distilled water. Then, the dispersion was taken in disposable folded capillary cells and was evaluated for zeta potential. Nano ZS zetasizer uses Smoluchowski equation for the calculation of zeta potential centered on the amount of doppler shift occurring due to electrophoretic mobility of colloidal particles in response to the electric field applied to the dispersion.

#### 2.3.3. % Entrapment Efficiency

Ref. [33] For the determination of entrapment efficiency, free FBX was separated from entrapped FBX in cubosomes by centrifuging it at 6000 rpm for a period of 15 min at a temperature of 25 °C using Remi Centrifuge. Then, supernatant of the centrifuge tube which contains cubosomal dispersion of FBX was separated carefully without disturbing the hard pallet of free drug, which was formed at the bottom of the centrifuge tube. Cubosomal dispersion of FBX was broken down using ACN:Methanol (9:1) for quantitative analysis of FBX. The absorbance of the prepared sample was then calculated using UV visible spectrophotometer at a wavelength of 315 nm. The % entrapment efficiency was calculated with the help of Equation (1).

(1) $$\%EE=\frac{Amount\;of\;entrapped\;drug}{Total\;drug\;added}\times 100$$

#### 2.3.4. Total Drug Content (% Assay)

For the determination of total drug content of the prepared formulation, 1 mL of the cubosomal dispersion, which was equivalent to 4 mg of FBX, was accurately withdrawn and was dissolved in 10 mL of ACN. The prepared samples of FBX were then analyzed using UV visible spectrophotometer at a wavelength of 315 nm. The % total drug content of cubosomes of FBX was calculated using Equation (2).

(2) $$\%Total\;drug\;content=\frac{Amount\;of\;total\;drug\;estimated\;}{Total\;drug\;added}\times 100$$

#### 2.3.5. Shape and Surface Morphology

Ref. [29] Transmission electron microscopy was employed for the evaluation of shape and surface morphology of the FBX-loaded cubosomes. For performing the test, the dispersion was smeared on a carbon-coated grid, and any extra material was removed and the grid was dried at room temperature for a period of 5 hrs. Transmission electron microscope (CM 200, Philips, Amsterdam, Netherlands) was employed with the following process parameters, i.e., the operating voltage was set in a range of 20–200 kV to visualize cubosomes at suitable magnification with an accelerating voltage of 20 kV.

#### 2.3.6. Small Angle X-rays Scattering

Ref. [29] Bruker Nanostar Xeuss 2.0 model was employed for conducting SAXS experiments furnished with a rotating anode and three-pinhole collimation. The device employs Cu-Kα radiation having a λ_max_ of 1.54 Å and a sample to detect a length of approximately 105 cm. Anode was set at 45 kV and 100 mA current. The samples were transferred in a 2 mm quartz capillary (from Charles-Supper, Westborough, MA, USA) having 10 μm wall thickness. For keeping reference, scattering from glassy carbon film was employed. The temperature of sample holder was maintained by Peltier unit. The obtained data was taken on a HISTAR gas filled multi-wire detector. Further, the 2D data was circularly averaged for the conversion of data to 1D. The scanning of samples was performed for a period enough to obtain at least two million counts. Further, these were normalized with the transmission coefficient of the sample and the acquisition time. The scattering emerging from silver behenate was employed for the calibration of Detector.

#### 2.3.7. Headspace Gas Chromatography (HS-GC) Testing for Residual Solvent

##### Standard Preparation

In 10 mL volumetric flask, 0.13 mL of ethanol equivalent to 0.1 g was taken and the final volume was made up to mark using DMF (dimethyl formamide) which gave final concentration of 10,000 ppm. In other 10 mL volumetric flask, 1 mL of above obtained solution was taken and final volume was made up to mark using deionized water to obtain final concentration of 1000 ppm [34].

##### Sample Preparation

A volume of formulation (0.105 mL) equivalent to 0.1 g was taken in 10 mL volumetric flask and the final volume was made up to the mark with DMF. From the above solution 1 mL was taken in 10 mL volumetric flask and volume was made up using deionized water. Sample was injected into column (capillary column: CR-624, Dimensions: 30 m, 0.53 mm, 3.00 µm) at 80 °C using nitrogen as carrier gas. Other parameters such as carrier gas flow rate, H_2_ gas flow rate, air flow rate, injection volume, injector temperature and detector temperature were set to 40 mL/min, 30 mL/min, 300 mL/min, 0.2 µL, 260 °C and 260 °C, respectively. Total run time was set at 20 min [34].

### 2.4. Characterization of MN Patch

#### 2.4.1. Axial Fracture Force

Brookfield CT3 texture analyzer was employed for the measurement of axial needle fracture force. For performing this, double sided adhesive tape was used for placing MN arrays on the texture analyzer’s mobile probe. This step was performed carefully in a manner that axis of MN aligns parallel with axis of mobile probe. After this, probe was automated for pressing the MNs on a rigid, flat steel [35]. During testing, the needle strength graph was made with the help of Texture Pro CT data acquisition software. An axial fracture force using following Equation (3) with the help of peak load obtained from the graph generated by Texture pro CT.

𝐹 *=* 𝑚𝑔 (3)

where: *m* = mass applied for breaking of MN. *g* = gravitational force.

#### 2.4.2. In Vitro Dissolution Study

##### Preparation of Gelatin Slab

Stratum corneum exhibits a water content of approx. 30 ± 5%. To simulate the conditions of stratum corneum, artificial gelatin skin was designed having a similar hydration level. This was achieved by adding 35% water and 65% gelatin. For preparing the artificial gelatin skin, 6.5 g of gelatin was taken and was transferred in 10 mL water and this solution was kept for hydration for a period of 30 min. Further, the gelatin was solubilized with the help of water bath at a temperature at 60 °C with continuous stirring. The resulting solution was transferred into a glass Petri dish and the water was permitted to vaporize till a weight of 10 g was attained. The resulting film obtained after evaporation was cut out into small square pieces.

##### In Vitro Dissolution Study

Optimized batch of MN patch was implanted in the gelatin film and removed at various time intervals (15 and 30 s, 1, 1.5, 2, 2.5, 3, 3.5, 4, 4.5, and 5 min) and viewed under microscope [36].

#### 2.4.3. Shape and Surface Morphology

For characterization of MN’s shape and their surface morphology, fast dissolving MN patches were affixed on sample stub and observed under JSM-5610LV scanning electron microscope (JEOL, Tokyo, Japan) wherein the accelerating voltage was set at 20 kV [36].

#### 2.4.4. Skin Penetrability

For checking the skin penetrability, double sided adhesive tapes were employed for mounting the MNs on mobile probe of Brookfield CT3 texture analyzer. Care was taken that the axis of MN was aligned parallel to the axis of the mobile probe. Probe was automated for pressing the MNs on the full thickness of pinned pig ear skin on a soft sponge pad under slight tension for simulating in situ mechanical support. The insertion speed of the moving probe was set at a speed of 20 mm/s. Skin area, on which MN was applied, was treated with trypan blue dye for a period of 5 min. A tissue paper was used for wiping off the excess dye from the skin. Further, digital camera was employed for taking the pictures of stained pores [37].

#### 2.4.5. Pore Closure Kinetic

For performing this study, rat skin was pinned with slight tension onto the soft board, for simulating in situ mechanical support. Five pieces of skin were used for the study and one patch was applied on each piece. At different time intervals, i.e., 0, 3, 6, 12 and 24 h, MN patch was detached from one piece of skin. The sections of epidermis were taken using cryo-microtome to observe for pore closure with the help of Eclipse H600L inverted microscope (Nikon, Tokyo, Japan) [37].

#### 2.4.6. Physical Stability of Cubosomes in Fast Dissolving MN Patch

After preparing the fast dissolving MN patch, instantly the physical stability of the cubosomes in MN patch was examined for vesicle size and entrapment efficiency. For this, the MN patch was solubilized in pre-filtered distilled water for obtaining the dispersion of cubosomes. The vesicle size and entrapment efficiency were estimated by the methods described above [37].

#### 2.4.7. Total Drug Content

Double distilled water was taken at a volume of 10 mL and used for solubilizing prepared MN patch of FBX. Then, 1 mL of prepared samples were diluted with acetonitrile: Methanol (9:1), respectively, and quantitatively analyzed using the UV spectrophotometer at λ_max_ of 315 nm [37].

### 2.5. In Vitro Drug Release Study

A dialysis membrane having molecular weight cut off in range of 12–14 K dalton in the Franz diffusion cell was employed for conducting in vitro drug release study. In case of Franz diffusion cell, the donor compartment has a volume capacity of 20 mL. To perform in vitro drug release, 30% ethanolic phosphate buffer pH 7.4 served as a diffusion medium [38]. For performing the study, plain drug suspension in water (1 mL), cubosomes of FBX (1 mL), MN patch containing plain FBX, and MN patch containing FBX cubosomes all equivalent to 3.4 mg were placed in the donor compartment. Further, from the receptor compartment, samples (1.0 mL) were removed at steady time intervals (0.5, 1, 2, 3, 4, 5, 6, 8, 12 and 24 h) and the identical volume (1.0 mL) was replaced by a fresh diffusion medium. Samples were evaluated using the UV spectrophotometer (Shimadzu, Kyoto, Japan, model: UV 1800) at λ_max_ of 315 nm. Triplicate readings of all experiments were recorded and further average of these readings was considered [36,39].

### 2.6. In Vitro Cell Viability Study

#### 2.6.1. Cell Culturing and Sub-Culturing

The cell culture of fibroblast 3T3 was bought from NCCS, Pune. The received flask was kept in an anaerobic incubator for a period of 24 h at a temperature of 37 °C and 5% CO_2_ without removing the media. Later, culture medium from the flasks was taken out and the adherent cells were washed with the help of PBS pH 7.4. Freshly prepared Trypsin-EDTA solution was then poured into the flask in order to completely cover the cell monolayer and was kept in the incubator for 2 min at 37 °C for detachment of adherent cells. For neutralizing trypsin’s activity in the flask, fresh growth medium was poured into the flask. Further, the cell culture was exposed to centrifugation at 1200 rpm for a period of 5 min. Then, after discarding the supernatant, resulting cells were re-suspended in a fresh growth medium. Cells were counted using neubauer counting chamber and transferred into new flasks at a plating density of 1 × 10^4^ cells/cm^2^. These flasks were kept in incubator set at a temperature of 37 °C and 5% CO_2_ to facilitate cell growth. The growth media was renewed every third day and passaging was conducted once the culture attained 80–90% confluency [40,41].

#### 2.6.2. MTT Assay

For the determination of safety, viability evaluation of fibroblast 3T3 cells was performed with the help of 3-(4,5-dimethylthiazol-2-yl)-2,5-diphenyltetrazolium bromide (MTT) assay [42]. Principle of this assay is based on the fact that mitochondrial dehydrogenase is responsible for the reduction of yellow-colored tetrazolium MTT whose production is found in viable (metabolically active) cells. The resultant intracellular purple formazan is solubilized and quantified with the help of spectrophotometer. Suspension of 3T3 cells in growth media was prepared from its culture using the same method as described above. A 96 well plate was used for seeding of the cells (5000 cells/well) and then it was kept in the incubator for a period of 24 h to facilitate cell growth and its attachment to the plate surface. After 24 h, growth media was discarded and 200 μL of fresh treatment media was transferred to these wells. Then, cubosomal dispersion of FBX and FBX suspension were diluted in growth media to obtain 1000 µg/mL of FBX. From these prepared cubosomal dispersions in fresh growth medium, 100 µL was added in different wells of 96 well plate. The plate was then incubated for 24 h and then the treatment media present was discarded. Then, 200 µL of growth media and 100 μL of MTT solution were transferred to each well and the plate was kept in the incubator for a period of 4 h. After this, 200 μL of dimethyl sulfoxide was transferred to each well for solubilizing formazan crystals, after removing growth media and MTT solution carefully. A microplate reader 690 XR from Bio-Rad, California, USA was utilized for the measurement of absorbance of the resultant solution. Measurement was performed at 570 nm. Cells viability in wells were treated with phosphate buffer saline pH 7.4, which acted as negative control, and was considered as 100% for the calculation of “% cell viability” [42].

### 2.7. In Vitro Permeation Study

MN patch of FBX, FBX cubosomes loaded MN, suspension of FBX and optimized cubosomes of FBX were tested for deposition profile and permeation with the help of full thickness rat abdominal skin. An abdominal area of rat was shaved to remove hair before harvesting the skin. A harvested rat skin was stored at −20 °C until it was needed for FBX permeation study. The evaluation was conducted by employing a Franz-type diffusion cell having a 20 mL receptor chamber. For performing this experiment, 30% ethanol solution prepared in distilled water was used for filling the receptor compartment and circular water bath was employed for maintaining its temperature at 37 °C. Before initiating the permeation experiment, the skin sections were thawed at room temperature. The skin sections were kept over a soft sponge pad and 30% *v*/*v* ethanol solution prepared in distilled water was used to impregnate the skin for a period of 30 min. This was performed for equilibration. Further, the skin sections were affixed between the receptor and donor compartment. Care was taken that the stratum corneum faces the donor compartment. Diffusion media used in the Franz diffusion cell was stirred at a speed of 100 rpm. After equilibration was achieved, cubosomal dispersion of FBX and FBX suspension (equivalent to 3.41 mg of FBX) was added in donor compartment. MN patch of FBX and cubosomes-loaded MN patch that had an identical amounts of drug were applied over the skin sections. This was achieved by the application of mild pressure using thumb on the skin which was kept under slight tension. Subsequently, it was affixed in place. From the sampling arm of the diffusion cell, samples that had a volume of 0.5 mL were taken at various time points, i.e., 0.5, 1, 1.5, 2, 3, 4, 5, 6, 7, 8, 12 and 24 h. Further, fresh diffusion media of the same volume were replaced in order to maintain the total volume. The skin section was removed from the Franz diffusion cell after 24 h and the skin was washed with 5 mL diffusion media three times. For calculation of the drug adhered to the skin, washings of the skin were saved. A scalpel was used for cutting the washed skin into small pieces. Then, these pieces were suspended in methanol, homogenized in cold conditions for a period of 5 min and then were sonicated using bath sonicator for a period of 15 min. For quantification of the drug accumulated in skin, the drug was removed by centrifuging it at an rpm of 5000 for a period of 10 min. All the samples were filtered with the help of 0.2 μm membrane filter and the quantification of the drug was performed by employing HPLC. The cumulative quantity of drug that permeated through the skin (per cm^2^ surface area of skin) was calculated. Finally, a graph was plotted with the concentration of the cumulative amount of drug permeated per cm^2^ surface area of skin against time. For transdermal steady flux (JSS; μg/cm^2^/h), a slop of the terminal portion of graph of concentration of FBX permeated across skin against time was found [37,43]. Equation (4) was used for the calculation of permeation enhancement ratio [44].

(4) $$PER=\frac{J_{SS}^{test}}{J_{SS}^{control}}$$

where $J_{SS}^{test}$ is steady state flux via test formulation and $J_{SS}^{control}$ is steady state flux via FBX suspension.

### 2.8. In Vitro Fluorescence Microscopy Study

With the help of fluorescence microscopy, permeation behavior of the formulations which were developed was illustrated. FITC suspension, its MN patch, optimized cubosomes and cubosomes (FITC loaded)-loaded MN patch were formulated and utilized for the study. FITC-loaded cubosomes and MN patch were prepared by replacing drug by FITC in optimized compositions. The rat skin was thawed at room temperature, equilibrated and fastened on Franz diffusion cell in the same way as explained in ex vivo skin permeation study. FITC-loaded formulations were smeared onto the stratum corneum layer of the skin in a similar way as explained in ex vivo skin permeation study. After a period of 12 h, skin sectioning was performed in dark environment using cryo-microtome, sections were fixed on a glass slide. Confocal laser scanning microscope was utilized for examining the fluorescence on the slide [37,45].

### 2.9. Histopathological Studies

Ref. [46] The study was conducted using rat abdominal skin, which was obtained from the sacrificed animals via academic protocol approved by the institutional animal ethics committee of the Maharaja Sayajirao University of Baroda (MSU/IAEC/2019–20/1902). Cubosomes-loaded MN patch of FBX, cubosomes of FBX and FBX drug suspension were applied on freshly excised rat abdominal skin. Apart from this, isopropyl alcohol (IPA) and PBS treated abdominal rat skin were used as positive control and negative control, respectively. After four hours, skins were immersed in 10% buffered formalin, dehydrated gradually increasing concentration of ethanol, immersed in xylene and finally embedded in paraffin. The 5-μm thick sections of skin were cut from these paraffin blocks using microtome and placed on glass slides. The paraffin wax was removed by gently warming the slides and washing the molten wax with xylene. Sections were then washed with absolute alcohol and water and stained with haematoxylin and eosin to determine gross histopathology. Commercial glycerol’s mounting fluid was used to finally mount the stained sections. Negative control and positive control slides were also prepared by treating rat skin with phosphate buffer solution pH 6.8 and isopropyl alcohol, respectively, using the same method. The slides were analyzed at 10-fold magnification using optical microscope [30,37].

### 2.10. In Vivo Pharmacokinetic Study

Sprague–Dawley rats weighing 200–270 g were procured from an official CPCSEA breeder. Rats which were obtained were placed in cages present in the animal house wherein the temperature was set at 22 ± 3 °C and light-dark cycle of fixed 12 h was maintained. Handling of the animals was performed with compliance to CPCSEA guidelines, Department of Animal Welfare, Government of India. Rats were kept on a standard chow diet and were given water as desired. In total, 30 rats were allocated to 5 groups randomly as shown in Table 2. Each group had two sets and each set had three animals. All group animals were fasted 12 h before starting the experiment. Marketed FBX tablet in a suspension form was administered to Group 1 animals through the oral route. Transdermal patch of FBX was applied on Group 2 animals. Group 3 animals were applied cubosomes of FBX, and MN patch of FBX was applied on Group 4 animals. Cubosomes-loaded MN patches were applied to Group 5 animals. Diethyl ether was employed as anesthetic agent while collecting the blood samples (not more than 0.5 mL) from retro orbital plexus. The collected blood samples were transferred to microcentrifuge tubes containing heparin at 1, 3, 5, 8, and 24 h from set-1 and 2, 4, 6, and 12 h from set-2 resulting 9 time points (1, 2, 3, 4, 5, 6, 8, 12, 24 h). The rats were replenished with saline solution. These blood samples were exposed to centrifugation at 3500 RPM for a period of 10 min at a temperature of 4 °C. The harvested samples of plasma were analyzed using a developed HPLC method at λ_max_ of 315 nm to estimate pharmacokinetic parameters such as C_max_, T_max_, T_1/2_, AUC and MRT [30,37,47,48].

### 2.11. Pharmacodynamic Study

Sprague–Dawley rats weighing 200–270 g were procured from an official CPCSEA breeder. Rats were handled in a similar manner as described in pharmacokinetic study (Section 2.10). Twelve rats were allocated to 4 groups randomly as shown in Table 3. All animals except Normal control (group 1) were sensitized with Potassium oxonate (PO) (250 mg/kg in 0.9% saline solution, intraperitoneally- IP) for induction of gout [49,50]. Initial paw volumes of rats were determined in all groups. Group 3 was treated with FBX (4.07 mg/kg orally) for 28 days as a standard control. Group 4 was treated with Cubosomes-loaded MN patch of FBX for 28 days. After 0 and 28th day, not more than 0.5 mL blood was withdrawn from retro-orbital plexus route. All animals were euthanized humanely using overdose of diethyl ether for assessing biochemical parameter (uric acid) and X-ray of rat paw [48].

### 2.12. Measurement of Uric Acid (UA)

When gout was induced in rat model, there was an increase in uric acid levels in rat blood which was measured quantitatively using uric acid enzyme kit which was purchased from Coral Clinical Systems, U.S. Nagar, Uttarakhand, India [51]. For measuring the concentration of uric acid, 3 test tubes were taken and labelled as blank, standard and sample. Then, 1.0 mL of working reagent (uricase) was added in each test tube and warmed at 37 °C for 5 min. After pre-warming, 25 µL sample (rat plasma) and standard (uric acid solution) were added in sample and standard test tube, respectively. In blank, only uricase solution was taken. All test tubes were incubated at 37 °C for minimum of 10 min. After 10 min, absorbance of all prepared samples was measured at 520 nm using UV spectrophotometer (Shimadzu, Japan UV-1800) against blank. A concentration of uric acid was measured using Equation (5).

(5) $$Uric\;Acid\;Concentration=\frac{Abs\;\left(sample\right)}{Abs\;\left(Standard\right)}\times Concentration\;standard\;\left(\mathrm{mg}/\mathrm{dL}\right)$$

### 2.13. X-ray

X-ray of rat’s paw was taken at Angela Lobo clinic, Vadodara, after harvesting it from the rat after euthanizing humanely to study change in bone shape after completion of pharmacodynamic study. X-rays of all animals from all groups were taken to study efficacy of the developed formulation.

### 2.14. Stability Study

Ref. [52] Stability study of the optimized Febuxostat-loaded cubosomes and MN patch of FBX cubosomes was performed as per the ICH guidelines of stability study. Three sample from the prepared optimized formulation were stored in airtight vials at room temperature (25–30 °C) and at (40–50 °C) in stability chamber. After time intervals of one, two and three months, cubosomes were analyzed for vesicle size and entrapment efficiency and MN patch of FBX cubosomes were evaluated for in vitro dissolution time and AFF (Axial Fracture Force).

## 3. Results and Discussion

The cubosomes of FBX were prepared using a bottom-up approach and then they were loaded in an MN patch to bypass the stratum corneum. The process and formulation parameters were optimized using the design of experiments. The mean vesicle size, PDI and zeta potential of the FBX cubosomes were found to be 157.5 nm, 0.165 and −17.2 mv, respectively. The vesicle size was small enough to penetrate the stratum corneum and further permeate to the blood vessels. According to the literature, if nanocarriers have a vesicle size below 300 nm, it can efficiently reach to the deeper layer of skin [53]. Here, cubosomes which have a vesicle size of less than 300 nm were successfully prepared. Due to the smaller size, they can efficiently reach the dermis layer of the skin, and can get absorbed in the systemic circulation to obtain the desired therapeutic concentration in the blood. Zeta potential most commonly indicates the stability of the colloidal formulation. Various components used in the preparation of colloidal dispersion contribute to the development of zeta potential on the vesicles. The optimum zeta potential required for the stability of colloidal dispersion is ± 30 mV according to various literatures [54]. The optimized cubosomes of FBX have zeta potential of −17 mV which is way below that required for the stability of cubosomal dispersion. The negative zeta potential was obtained due to the presence of free oleic acid in the lipid. However, the prepared cubosomal dispersion was found to be stable at room temperature due to the stealthing effect of the stabilizer (PVA) used in the preparation of cubosomes of FBX [55]. Moreover, the optimized cubosomes were not going to be stored in the dispersion form but were loaded into MNs, which are solid structures. Thus, zeta potential of −17 mv did not affect the physical stability of cubosomes in the MNs. The mean % entrapment efficiency of the optimized formulation was found to be 85.2%, while the total drug content of cubosomes was found to be 97.28%, i.e., 1 mL of cubosomal dispersion contained 3.89 mg FBX. In the case of entrapment efficiency, high efficiency and the drug loading of FBX was obtained in cubosomes. The lipophilic property of the entrapped FBX is responsible for the high % of entrapment efficiency of the optimized formulation (Febuxostat- log *p* value- 3.3). Additionally, cubosomes have a distinct advantage of providing high entrapment efficiency of the encapsulated drug according to the literature [27,56].

**Figure 1:**
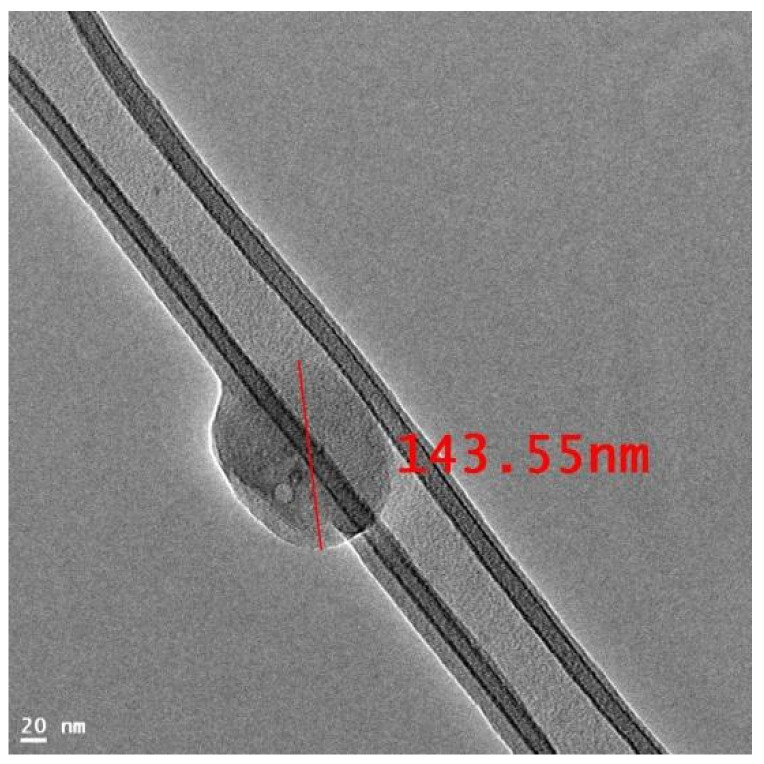
TEM image of Febuxostat-loaded cubosomes. From the TEM image of FBX-loaded cubosomes it can be observed that it has cubical shape.

The TEM image of the optimized FBX cubosomes is shown in Figure 1. The TEM image of cubosomes of FBX indicate that the cubosomes have a cubical shape with smooth surface [57]. The size of the cubosomes seen in the image was found to be in line with the results of vesicle size data obtained by the Malvern Zetasizer.

**Figure 2:**
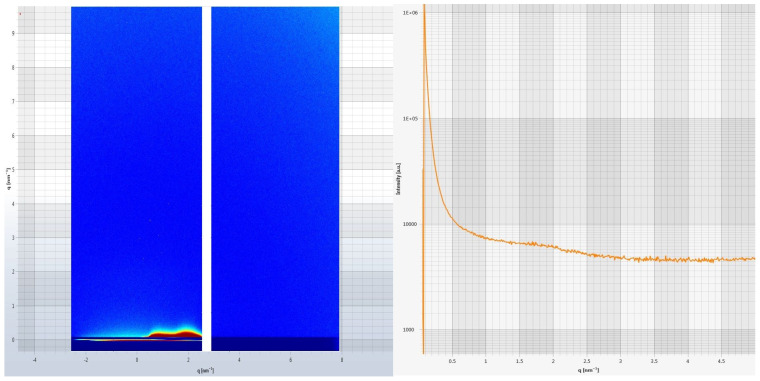
Scattering pattern of optimized cubosomes of FBX. SAXS analysis of prepared formulation was conducted to investigate the liquid crystalline structure of cubosomes. The diffuse scattering pattern of low intensity in the region of 1.0–2.25 A^−1^ indicating presence of water channels inside cubosomes which is a unique feature among all nanocarriers.

SAXS was used for the investigation of the liquid crystalline structure of the prepared cubosomes and the results are shown in Figure 2. It showed a sequence of two well-defined scattering patterns and one diffuse diffraction pattern at Q values of 0.12, 1.75 and 1.0–2.25 A^−1^ region with relative positions on a curve, respectively. The peak at the Q value of 0.12 A^−1^ indicates characteristic scattering peaks due to the cubic phase, whereas the peak at 1.75 A^−1^ reveals a scattering pattern due to the whole cubosome liquid crystalline structure. The key characteristic of this X-Ray scattering diagram was a diffuse scattering pattern of low intensity in the region of 1.0–2.25 A^−1^ indicating the presence of water channels inside cubosomes which is a unique feature among all nanocarriers [58].

Ethanol was used in the preparation of the cubosomal dispersion and hence the final product was evaluated for the presence of ethanol using HS-GC. The results indicated that 167.55 pm of ethanol was present in the final formulation. The presence of organic solvents in the final formulation may pose the risk of toxicity. In order to prepare the cubosomes, ethanol was used as a co-solvent. The limit of ethanol in the final formulation is 1500 ppm according to the ICH guidelines Q3C (R6) for residual solvents [59]. The ethanol content in the prepared cubosomes of FBX was within permitted concentrations as per ICH guideline Q3C (R6) indicating no risk of toxicity due to the presence of ethanol [59].

The optimized cubosomal dispersion was then used to formulate the fast dissolving MN patch. Characterization of the MN patch was performed for various parameters. The Axial fracture force (AFF) is a good indicator of the mechanical strength of MNs and thus it was determined. The MNs should have sufficient mechanical strength, i.e., 0.03 N/MN so that it can breach the stratum corneum without breaking and deliver the loaded carrier systems directly to the dermis layer of skin [60]. The high value of Axial fracture force (1.2 N-calculated from Supplementary Figure S1) indicates the sufficient mechanical strength of the developed MN patch of FBX. The MNs with low mechanical strength may break during application. From the dermis layer, FBX can be absorbed directly in the systemic circulation and inhibit the xanthine oxidase enzyme present in blood pool. This enzyme is responsible for the formation of uric acid [1]. Thus, it can be interpreted that the prepared MN patch of cubosomes of FBX has a required minimum mechanical strength according to the literature [60].

The observation of the in vitro dissolution time of MNs indicates that MNs start to dissolve immediately and complete dissolution is obtained in 1.25 min (Supplementary Figure S2). The intention in the present research work was to develop a fast dissolving MN patch in order to obtain a rapid drug release.

**Figure 3:**
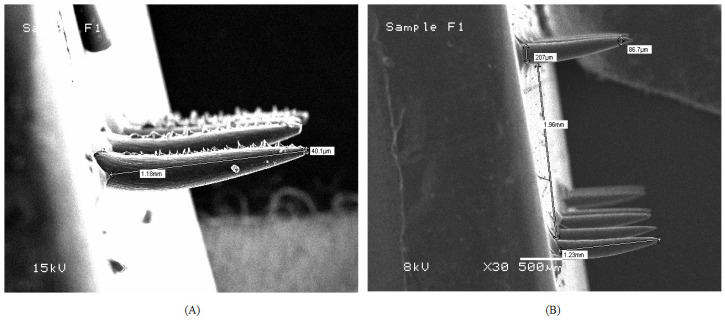
SEM of MN patch (**A**) MN patch of FBX-plain drug (**B**) MN patch loaded with cubosomes of FBX. SEM analysis was performed to analyse the difference between prepared MN patches.

SEM images of a fast dissolving MN patch are presented in Figure 3. These images showed smooth surfaced, conical MNs that have a length of 1.5 mm, and a base diameter of approx. 200 μm. The prepared MNs of cubosomes loaded with FBX and MNs of pure FBX were analyzed for SEM (Scanning Electron Microscopy). From the SEM images it was found that FBX cubosomes-loaded MNs have a smooth surface, while MNs of pure FBX have a rough surface. The rough surface of MNs of FBX was obtained due the insolubilized FBX. While cubosomes of FBX remained in a dispersed form and provided a smooth texture to the prepared MNs.

Skin penetrability of the prepared MN patches containing cubosomes of FBX was performed and the presence of tiny blue stains on the rat skin proves that the prepared MN patches were able to create the pores in the skin and deliver FBX directly to the dermis layer. (Supplementary Figure S3).

**Figure 4:**
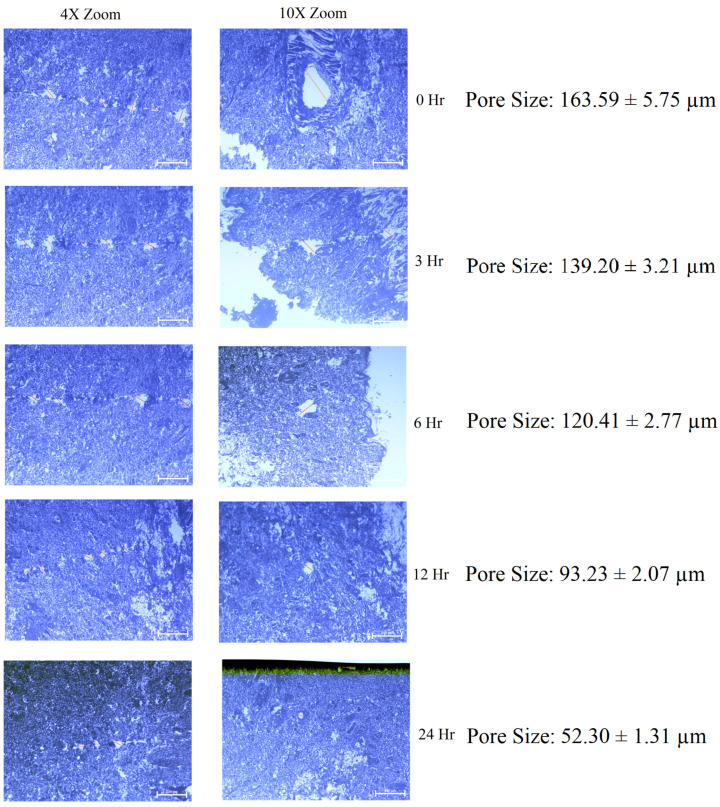
Pore closure kinetics for MN patch loaded with cubosomes of FBX. Pore closure kinetic study was performed to find out that how long the prepared pores will remain open after applying MN patch.

The application of an MN patch on rat skin formed pores which were detected using a microscope (4× and 10×) and its average pore diameters were measured as shown in Figure 4. From pore closure kinetic studies, it was found that the size of the micropores formed in the skin decreased with time and complete pore closure was observed in 24 h. The results are in concurrence with the reported literature [61]. Closure of the pores created due to the MNs is necessary in order to avoid the chances of infection resulting from the open pores.

The physical stability of the cubosomes in the MN patch was ensured by the determination of the particle size and entrapment efficiency of cubosomes after a complete dissolution of MN patch in water. The initial vesicle size and entrapment efficiency of the cubosomes of FBX were found to be 157.5 ± 4.16 nm and 85.2 ± 2.68%, respectively, while the vesicle size and entrapment efficiency after the dissolution of the MN patch were found to be 154 ± 3.94 nm and 84.9 ± 1.88%, respectively. No significant change in the vesicle size and entrapment efficiency of cubosomes of FBX was observed after loading them in the MN patch, suggesting its stability in the MN patch. The total drug content of the MN patch containing FBX cubosomes was found to be 98.14% which means each MN patch of FBX cubosomes contains 3.336 mg of FBX.

### 3.1. In Vitro Drug Release Study

**Figure 5:**
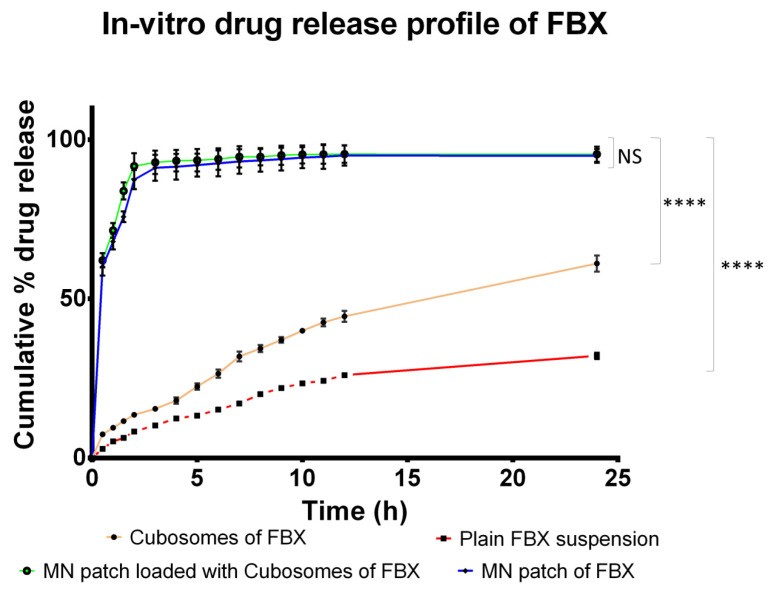
In vitro drug release from various developed formulations of FBX. In vitro drug release study was performed to find out the release time of FBX from various formulations. Each release study was performed in triplicate and SD was also included in figure in horizontal bar. NS indicates Not significant while **** indicates significance between data with *p* value < 0.0001.

The cumulative percent release of the drug from all four formulations at various time intervals are shown in Figure 5. Various mathematical models were applied to the data of drug release from all formulations to find out the release behavior of FBX and the results are listed in Table 4. The results of the in vitro release study showed that more than 60% of FBX was released from cubosomes in 24 h, which was significantly greater than the release from plain drug suspension (39.97%) probably due to its low solubility in phosphate buffer pH 7.4. FBX is practically insoluble in water. Thus, when its suspension was prepared in phosphate buffer pH 7.4 and filled in a diffusion bag, it was not able to solubilize. In order to permeate across the dialysis bag, the drug molecules must be present in a solubilized state. Thus, due to the poor solubility of FBX, only a limited amount of the drug was able to permeate through the dialysis bag. On the other hand, cubosomes have the advantage of improving the surface area which is in contact with the phosphate buffer pH 7.4. Therefore, the more the amount of the drug dissolved in a phosphate buffer pH 7.4 diffuses to the donor compartment. Due to this reason, a higher amount of FBX was able to permeate the diffusion membrane and a higher in vitro release of FBX was obtained in the case of cubosomes of FBX [62,63]. The highest drug release was obtained from MN loaded either with the plain drug or with cubosomes of FBX. The micropores formed in the dialysis membrane seem to be responsible for allowing higher amounts of the drug to permeate through the membrane. Various mathematical models were applied for the in vitro drug release study. In the case of cubosomes, the highest R^2^ value was obtained for the Korsmeyer–Peppas model suggesting a diffusion-controlled system, where the release rate was dependent on the drug concentration remaining within the cubosomes. Moreover, the n value of 0.607 for the Korsmeyer–Peppas model suggests non-Fickian diffusion of the drug from cubosomes [64]. Non-Fickian diffusion means that the diffusion of the drug from the cubosomes does not follow Fick’s laws of diffusion. However, in the case of MN loaded with FBX cubosomes, the highest R^2^ value was obtained for the first order indicating that the drug release rate depends on the concentration gradient across the membrane.

An in vitro release study of MNs of FBX and MNs loaded with cubosomes of FBX shows that more than 90% of the drug released in 2 h from both. The results showed a significant increase in drug release when compared with the drug release profile of FBX cubosomes. The optimized MN patches were able to penetrate the diffusion bag due to the sharp MN structure. After penetration in the membrane, MN structures rapidly dissolved due to the use of fast dissolving polymers leading to the rapid release of FBX in the diffusion medium. Whereas in the case of cubosomes, the drug has to diffuse through the intact diffusion bag and thus release is slow. Such observations suggest that due to the penetrating ability of MNs, a highly significant increase in drug release was possible. A Bonferroni’s multiple comparison test of two-way ANOVA was applied in between the column and relative symbology was marked in Figure 5. Based on this, it can be said that there is a significant difference between the release of FBX from cubosomes and FBX suspension compared with the MN patch loaded with cubosomes of FBX while there is no significant difference between the MN patch of FBX and the MN patch loaded with cubosomes of FBX. Various mathematical models were applied for the in vitro release study of both MN patches and, according to the R^2^ value for first order model, it was found to be highest which suggest that the release rate is dependent on the drug concentration in the carrier.

### 3.2. In Vitro Cell Viability Study

**Figure 6:**
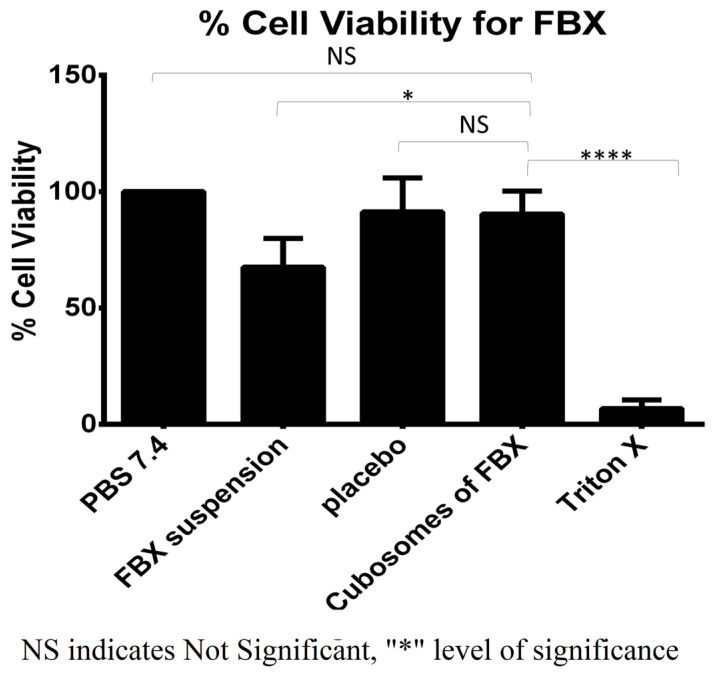
% viability of fibroblast 3T3 cells after treatment with cubosomes of Febuxostat. Cell viability study was performed to investigate the toxicity of prepared formulation on fibroblast 3T3 cells. This study was performed in triplicate. NS indicates Not significant while **** indicates significance between data with *p* value < 0.0001.

The cell viability data for FBX formulations are summarized graphically in Figure 6. In the cell viability study, a significantly lower viability of cells treated with Triton X 100 (6.82 ± 3.65%) indicated the validity of the positive control. The viability of cells treated with FBX-loaded cubosomes (90.32 ± 9.93%) was found to be significantly higher than the positive control and near to the negative control. A Dunnett’s multiple comparison test of two-way ANOVA between the column was applied to prove the significance difference between the columns. There is significance difference among FBX suspension and triton X compared with cubosomes of FBX while there is no significant difference among PBS 7.4 and the placebo compared to cubosomes of FBX. Moreover, the viability of cells treated with FBX suspension was 67.48 ± 12.36% which is significantly less than the cubosomes of FBX. This indicated a less toxic nature of developed formulations compared with the FBX suspension and triton X 100.

### 3.3. In Vitro Permeation Study

**Figure 7:**
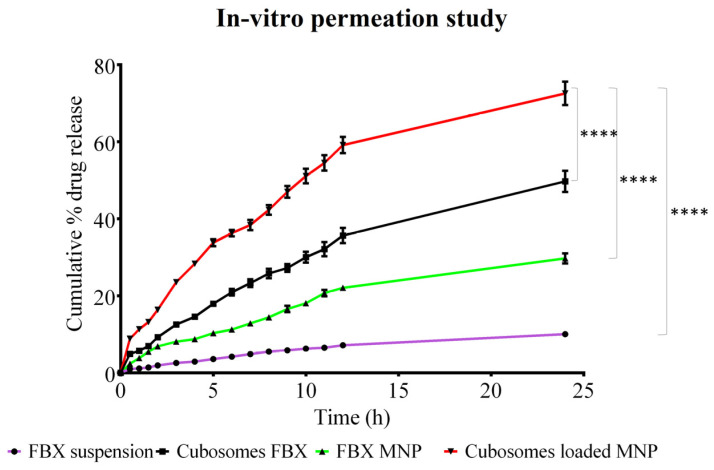
In vitro permeation of FBX from various developed formulations using rat skin. In vitro permeation was performed to investigate that the prepared formulations were able to cross the stratum corneum or not and release the drug to the release media. NS indicates Not significant while **** indicates significance between data with *p* value < 0.0001.

**Figure 8:**
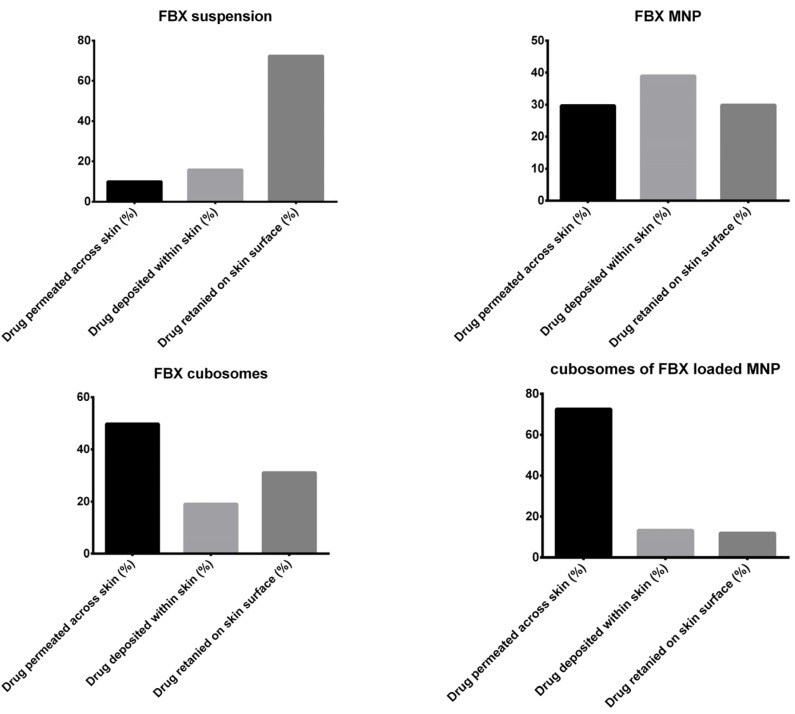
FBX distribution profile after 24 h of permeation study. During in vitro permeation study, the fraction of drug retained on skin surface, drug deposited within skin and drug permeated across the skin could be understood from this figure.

The results of the in vitro permeation studies through the full-thickness rat skin are shown in Table 5 and Figure 7. A Bonferroni’s multiple comparison test of two-way ANOVA was applied in between the column and the relative symbology was marked in Figure 7. Based on this, it can be said that there is a significant difference between the permeation of the drug across the skin from the cubosomes of FBX, the MN patch of FBX and FBX suspension compared with the MN patch loaded with cubosomes of FBX. A slight improvement in FBX permeation was observed with the MN patch of FBX (J_ss_—6.45 μg/cm^2^/h) as compared with FBX suspension (J_ss_—4.20 μg/cm^2^/h) due to the MN’s ability to permeate the skin barrier. However, in the case of the cubosomes of FBX-loaded MN patch, an 8.34-fold increase in permeation (J_ss_—35.06 μg/cm^2^/h, PER—8.34) was observed due to the microporation of the skin. The results reflected that cubosomes of FBX (J_ss_—18.43 μg/cm^2^/h) can permeate the skin barrier less significantly than the cubosomes of FBX-loaded MN patch. The amount of the drug retained on the surface and deposited within the skin was determined after the completion of release experiments and the data are presented in Figure 8. On the basis of the results of permeability from various formulations, they are organized in order of increasing permeability: FBX Suspension < FBX MN patch < FBX cubosomes < cubosomes of FBX-loaded MN Patch. In the case of the MN patch, permeation obtained through rat skin was less than through FBX cubosomes and MN patch loaded with cubosomes of FBX due to the very poor solubility of FBX in water. The synergistic effect of microporation on cubosome’s permeability was established as a significant enhancement was observed in the permeability through the cubosomes of FBX-loaded MN patch [37,65]. From Figure 8, it has been noted that that FBX MN exhibits the maximum deposition of FBX. The reason for this finding may be the lipophilic nature of FBX, which may not have been able to permeate to the systemic circulation in sufficient concentrations due to the deposition in the hydrophilic dermis layer. It can be concluded that permeation was better with the cubosomes of FBX-loaded MN when compared with cubosomes of FBX. In case of cubosomes of FBX, the retention of FBX on the skin was more compared with the cubosomes of FBX-loaded MN. Moreover, in the case of drug suspension, the maximum retention of the drug on the skin suggests that the drug alone is unable to cross the skin barrier efficiently.

### 3.4. Ex Vivo Fluorescence Microscopy Study

**Figure 9:**
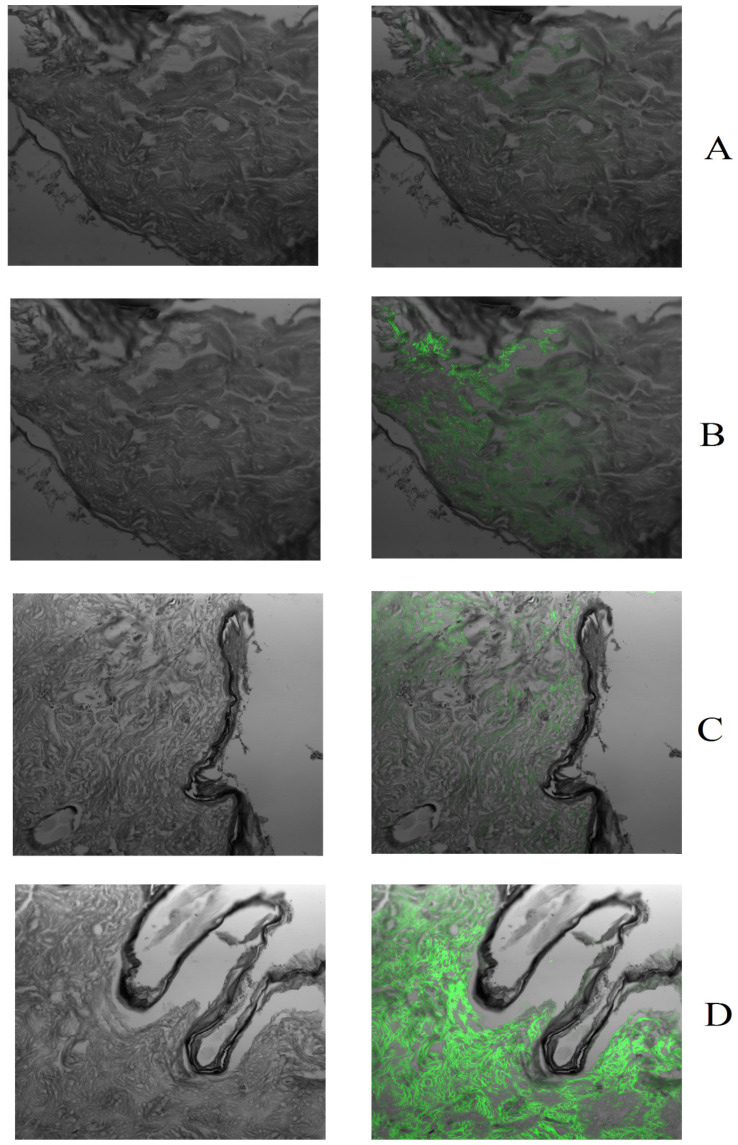
Fluorescence microscopic images of rat skin sections after 12 h of treatment with (**A**) FITC suspension, (**B**) MN patch of FITC, (**C**) cubosomes loaded with FITC, (**D**) MN patch with FITC cubosomes. From this figure, the permeation of drug across the skin from various formulations was well understood.

Sections of the rat skin were exposed to FITC-loaded formulations for a period of 12 h. Further, after exposure, fluorescence microscopic images of rat skin sections were taken and the images are presented in Figure 9. Based on the results of the ex vivo fluorescence microscopy study, formulations are organized in increasing order of the fluorescence: FITC Suspension < FITC MN patch < FITC cubosomes < MN patch loaded with cubosomes of FITC. It was noted that the data collected from the fluorescence microscope experiment complied with the ex vivo permeation and deposition data wherein maximum fluorescence was reported in sections of skin which were exposed to the MN patch loaded with FITC cubosomes. Therefore, it can be concluded that there is enhanced permeation through developed nanocarriers-loaded fast dissolving MN patches [37].

### 3.5. Histopathology Studies

**Figure 10:**
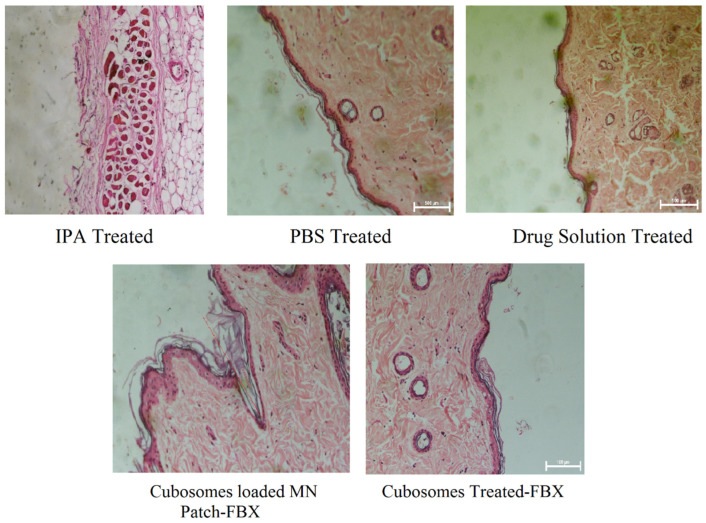
Histopathology study of developed formulation. Histopathology study was performed to understand the toxicity various formulation on the skin using positive and negative control.

The haematoxylin and eosin-stained sections of rat abdominal skins treated with developed cubosomes of FBX and cubosomes-loaded MNs of FBX were examined under a microscope for any pathological changes and compared with negative (PBS 7.4) and positive controls (IPA) to study the safety aspects of using an MN patch. The microscopic images have been shown in Figure 10. The sections of skins treated with developed cubosomes of FBX and cubosomes-loaded MNs of FBX showed almost similar cellular integrity as compared to skin treated with phosphate buffer saline (pH 7.4) as a negative control with no sign of inflammation. The section of skin treated with isopropyl alcohol as a positive control showed considerable damage to skin layers as an indication of irritation and toxicity. Therefore, it can be concluded that there is no evidence of damage to the skin samples after treatment with FBX suspension, cubosomes of FBX and MN loaded with cubosomes of FBX. This finding proves that the prepared formulations and drug samples are non-toxic and non-irritant to the skin samples. Moreover, in the case of skin sections treated with MN, microchannels were observed, which were created due to the insertion of the MN. This proves that MNs can effectively bypass the stratum corneum and deliver the drug directly to the dermis layer of skin for systemic delivery of the drug.

### 3.6. Pharmacokinetic Study

**Figure 11:**
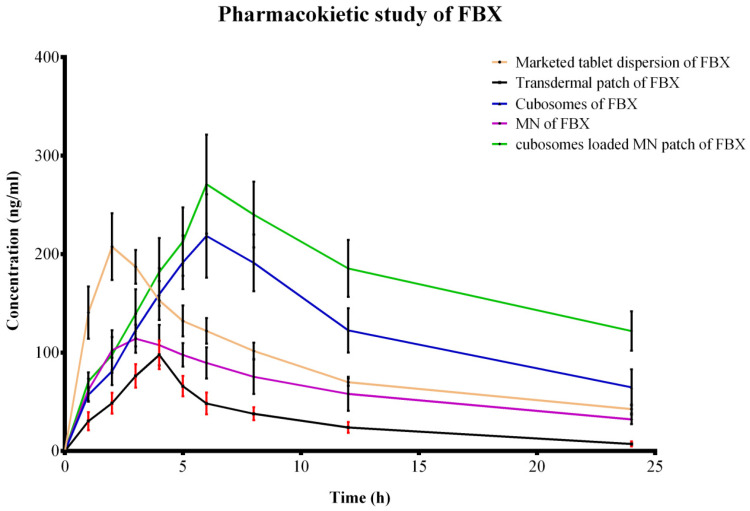
Plasma FBX concentration vs. Time profile of various dosage forms in Sprague–Dawley rats. Pharmacokinetic study was performed to understand the absorption of FBX from various formulations. Drug absorption profiles of various formulation were also compared using this figure.

HPLC was employed for the determination of concentrations of FBX in the blood plasma of rats and the data are represented graphically in Figure 11. Thermo Scientific™ Kinetica Software was utilized for the calculation of various PK parameters from the collected data and is summarized in Table 6.

The results shown in Table 6 indicate that in the case of marketed oral tablets in suspension form, high C_max_ and low t_max_ values are obtained which indicate the rapid and good absorption of the drug through the oral route. Moreover, the value of T_1/2_ and MRT indicate the slow elimination from the body leading to BID (twice a day) administration. In the case of simple transdermal film, very low values of C_max_ are obtained indicating much less absorption of the drug through the transdermal route in the absence of any penetration enhancement. On the other hand, cubosomal gel, FBX MN and FBX cubosomal MN show significantly higher values of C_max_ indicating an enhanced permeation of FBX compared with the transdermal film of FBX. Apart from this, cubosomal gel and FBX cubosomal MNs achieved C_max_ comparable to the marketed oral tablet and FBX cubosomal MNs obtained the highest C_max_ (271.03 ng/mL). The comparison of the AUC_0–t_ values indicate a highly significant difference between the different groups and the maximum value was obtained from FBX cubosome MNs. This is due to the synergistic effect of the cubosomes and the MN in enhancing the skin permeation. Both the approaches, viz., cubosomes and MNs individually led to an increase in transdermal permeation. However, FBX cubosomal MNs show a maximum AUC_0-t_ and MRT suggesting the maximum absorption of FBX and FBX is available in systemic circulation for a maximum period of time compared with other all formulations. However, a combination of both approaches results in a synergistic effect and an almost three-fold enhancement in bioavailability was observed. The results are in concurrence with the earlier reports [37]. An increase in the bioavailability can lead to a reduction in the dose and the associated side effects making the treatment safer and more patient compliant. The higher values of T_1/2_ and MRT also indicate the possibility of a reduction in the dosage frequency. Upon the release of the FBX cubosomes after the MNs dissolution in the skin, the drug is released in a sustained manner from the cubosomes. The results are in concurrence with the in vitro release studies which indicate a sustained release of the drug from cubosomes.

### 3.7. Pharmacodynamic Study

**Figure 12:**
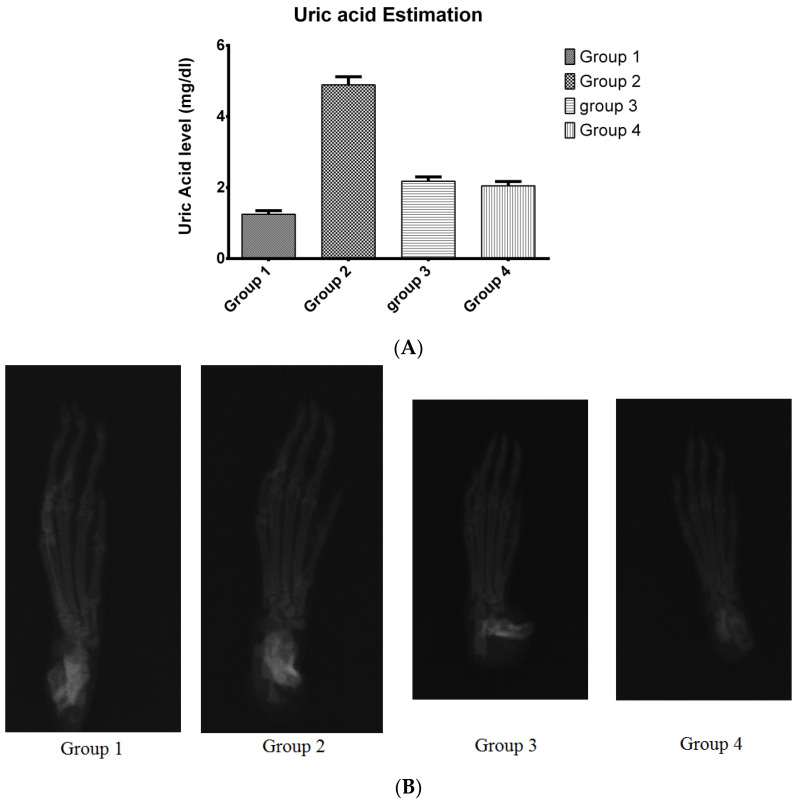
Pharmacodynamic study (**A**) Measurement of serum uric acid level in rat serum, (**B**) X-ray of rat’s paw to study efficacy of the developed formulation of FBX. The efficacy of the prepared formulation was compared to the marketed formulation by its ability to reduce the serum uric acid level.

As shown in Figure 12, there was an increase in uric acid levels in rat blood in the animals in which an induction of gout was performed. As shown in Figure 12A, the standard and test control animal groups have lower blood uric acid than the model control group indicating the efficacy of the developed formulation.

The bone X-rays of rats from all groups are depicted in Figure 12B. From Figure 12B, it was observed that there was no tophi formation in any set of the animal groups. Moreover, no structural change in bones of rat was observed during the study.

In the pharmacodynamic study, gout was induced in the Wistar rat using potassium oxonate. In the rat, uricase enzymes are responsible for the metabolism of uric acid. Thus, to increase uric acid levels in rat blood, uricase enzymes must be inhibited. Potassium oxonate inhibits this uricase enzyme and increases uric acid levels in rat blood [66]. When the blood levels of uric acid increases above 6 mg/dL, it results in the formation of Monosodium urate (MSU) crystals. These MSU crystals then deposit in various joints and form a tophus-like structure. However, these elevated levels of uric acid do not result in the formation of MSU crystals every time. Thus, in many cases the hyperuricemia patients do not develop this tophus-like structure. However, these patients are prone to the formation of tophi around various bone joints if uric acid levels in blood are not controlled [1].

Due to this reason, bone X-rays of rats did not confirm the formation of any tophi during the induction of gout. However, increased levels of uric acid suggests the induction of hyperuricemia, and this increased level of uric acid in the blood is responsible for the induction of gout according to the literature [1,67,68]. The uric acid level was controlled using a developed and marketed formulation and proved the efficacy of the MN patch loaded with cubosomes of FBX and cubosomal gel of FBX.

### 3.8. Stability Study

**Figure 13:**
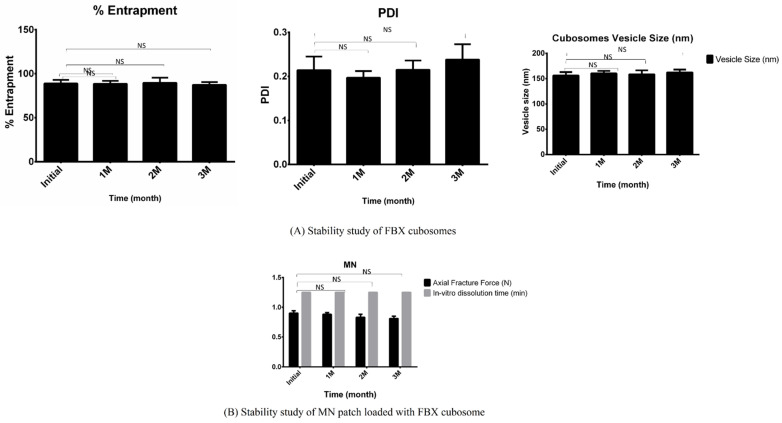
Stability Study of developed formulations. Short term stability study of the prepared formulations was conducted and they were characterized for various tests such as vesicle size, PDI, % entrapment efficiency for FBX cubosomes and AFF (Axial Fracture Force) and in vitro dissolution time for FBX cubosomes of MN patch. NS indicates Not significant.

The results of AFF (Axial Fracture Force) and the in vitro dissolution time of MNP and vesicle size, PDI and the percent of drug entrapment of cubosomes stored for up to three months are summarized in Figure 13. In the stability study it was found out that, under storage conditions, AFF of the MN patch was slightly decreased while there is no effect on the in vitro dissolution time of developed MNP. Similarly, a slight increase in vesicle size and PDI, as well as a slight decrease in drug entrapment, was evident in the storage in cubosomal formulations. However, the values observed after three months were found to be within the desirable limits required for formulations to perform effectively. Such observations at intermediate temperatures and a high relative humidity could interpret that solid MN patches should be stored in airtight containers with silica bags to absorb moisture content while cubosomal dispersion should be stored in airtight containers at room temperature. Bonferroni’s multiple comparison test of two-way ANOVA was applied in between the column and relative symbology was marked in Figure 13. From this, it can be concluded that there is no significant difference between the obtained data sets meaning the developed formulations are stable in test conditions.

During the development of cubosomal FBX MN, processes that ease up the lab process to scale up to commercial scale is necessary. For instance, cubosomes preparation using a bottom-up approach, MN patch preparation by a micromold casting method, etc. However, the scale up for cubosomes and MN preparation still needs to be investigated. To ensure patient concern due to deposition of polymers used to prepare polymeric MN needs to be investigated. Like other polymeric MNs, cubosomal FBX MNs were also anticipated to deposit small amounts of polymers with every application which may further enhance upon several uses over an extended period of time. However, the molecular weights of these polymers play a significant role in their elimination. For example, a report on the safety assessment of PVP suggests that for its complete excretion, the polymer size must be below the glomerular threshold [69,70]. Hence, while selecting the polymers, consideration was given to low molecular weight polymers, viz., poly vinyl pyrollidone (MW, 3.5 kD) and poly vinyl alcohol (MW, 6 kD). Additionally, rotating the site of application may also be helpful in avoiding local accumulation. Thus, polymer accumulation during multiple uses of MNs still needs to be investigated. Skin often repairs itself against minor mechanical trauma associated with injuries such as scrapes and scratches without developing infection. Hence, despite the breaching of the stratum corneum to deliver the payload to viable epidermis and dermis, MNP poses less risk of infection via microbial influx through MN-induced microchannels as experienced during their appropriate clinical usage [71]. Further, an in vitro study conducted to evaluate the ability of microorganisms to cause infection when allowed to ingress via MN-induced microchannels revealed that no microorganisms were able to penetrate viable epidermis and dermis making it unlikely to develop infections in immunecompetent patients [72]. Therefore, it still remains for the present study to prove that microorganisms are not able to cross the microchannels that are created by MNs. However, in order to assure safe use in every patient, the need for sterility in MNPs has been widely discussed and seems to be critical for regulatory approval [73]. Aseptic manufacturing as well as terminal sterilization via moist heat, dry heat and gamma radiation methods have already been investigated [74]. Aseptic manufacturing is inconvenient and fairly expensive while terminal sterilization often adversely affects the physicochemical properties of polymeric MNPs or their cargo. Hence, a suitable sterilization method must be considered carefully to avoid such issues where the use of antimicrobial agents has also been suggested to achieve a self-sterilization effect at the injection site [73,75]. Furthermore, being fast dissolving in nature, cubosomal FBX MNs will not produce any sharp waste and, hence, will not require safe disposal.

## 4. Conclusions

The present investigation was aimed to overcome the poor oral bioavailability and gastrointestinal-related disturbances associated with the FBX via the development of a transdermal formulation which can deliver FBX in a controlled manner to achieve therapeutic goals with better patient compliance. In the present study, in the first phase, cubosomes of FBX were prepared with GMO as a lipid and PVA as a stabilizer using a bottom-up approach and were evaluated for various characterization tests. Then, in the next phase, the developed cubosomes of FBX were loaded in the MN patch and evaluated for various characterization tests. The QbD methodology was applied for the optimization of both formulations. The particle size and entrapment efficiency were selected as CQAs for cubosomes of FBX while the axial fracture force and dissolution time of MNs were selected as CQAs for FBX cubosomal MNs. The cubosomal FBX and FBX cubosomal MNs were subjected to the stability study and were found to be stable during the stability period. However, the MN patch required special airtight container closure systems due to the hydroscopic nature of a polymer used in preparation of the MN patch. Form the ex vivo study, it can be concluded that the MN patch loaded with cubosomes of FBX has the highest transdermal flux followed by cubosomes of FBX. From the histopathological evolution of the prepared formulations, it can be established that the prepared formulations are nontoxic to the rat skin and both MN patches are capable of breaching the stratum corneum successfully. From the pharmacokinetic study, it can be concluded that the MN patch loaded with cubosomes of FBX can permeate the stratum corneum and deliver the FBX to systemic circulation more successfully than any other formulation, followed by the cubosomal gel of FBX. In the case of the pharmacodynamic study, it was observed that the MN patch loaded with cubosomes of FBX was able to control the uric levels in rat blood more successfully than other formulations, followed by the cubosomal gel of FBX.

## Figures and Tables

**Table 1:** Composition of optimized formulation.

**For Cubosomes of FBX**	
Conc. of GMO * (%*w/v*)	Conc. of PVA ** (%*w*/*v*)
7.6	1.2
**For MN patch of cubosomes of FBX**	
Conc. of PVA ** (%*w/v*)	**Conc. of lactose** (%*w*/*v*)
33.46	8.7

* GMO: Glyceryl monooleate. ** PVA: Polyvinyl alcohol 6000.

**Table 2:** Animal grouping for pharmacokinetic study of FBX.

Sr. No.	Groups		
	Treatment	Set-I	Set-II
1	Marketed oral suspension of FBX (4.07 mg/kg)	3 *	3 *
2	Transdermal patch of FBX (4.07 mg/kg)	3 *	3 *
3	Developed cubosomes of FBX(4.07 mg/kg)	3 *	3 *
4	MN patch of FBX (4.07 mg/kg)	3 *	3 *
5	Cubosomes of FBX loaded MN patch (4.07 mg/kg)	3 *	3 *
**Total**		**30**	

* Not sacrificed, rehabilitated and used in pharmacodynamic study after washing period.

**Table 3:** Animal grouping for pharmacodynamic study of FBX.

Sr. No.	Group	Treatment	No of animals
1	Normal control	Distilled water	3 **
2	Model control	PO * (250 mg/kg in 0.9% saline solution, Intraperitoneally-IP) [49,50]	3 **
3	Standard control	FBX (4.07 mg/kg orally) + PO (250 mg/kg in 0.9% saline solution, Intraperitoneally-IP) [49,50]	3 **
4	Test control I	Cubosomes-loaded MN patch of FBX (4.07 mg/kg transdermally) + PO (250 mg/kg in 0.9% saline solution, Intraperitoneally) [49,50]	3 **
**Total**			12 **

* PO: Potassium Oxonate. ** Sacrificed to harvest rat paw for X-ray.

**Table 4:** Various mathematical models and their correlation coefficient values.

Mathematical Models		MN Patch of FBX	MN Patch of FBX Cubosomes	Cubosomes of FBX	FBX Suspension
**Zero order**	**R^2^**	0.6601	0.5432	0.7267	0.3063
**First order**		0.9719	0.9801	0.9498	0.5742
**Higuchi**		0.7631	0.7074	0.9568	0.9608
**Hixon–Crowell**		0.8492	0.7902	0.9114	0.4905
**Korsmeyer–Peppas**		0.8778	0.8339	0.9813	0.9653
	**N**	0.104	0.088	0.607	0.465

**Table 5:** Amount of FBX permeated across Rat skin.

Time	Amount of Drug Permeated Per Unit Area of Rat Skin (µg/cm^2^)			
	FBX Suspension	FBX Cubosomes	FBX MNP	Cubosomes Loaded MNP
0.5	7.10 ± 0.42	34.20 ± 0.90	16.41 ± 0.76	61.49 ± 1.07
1.0	7.86 ± 0.31	39.68 ± 0.37	26.54 ± 0.63	78.60 ± 1.38
1.5	9.74 ± 0.66	48.13 ± 0.69	38.12 ± 1.41	91.56 ± 1.92
2.0	13.50 ± 0.88	64.21 ± 1.10	47.66 ± 5.22	113.67 ± 0.89
3.0	17.89 ± 0.30	87.16 ± 2.79	56.23 ± 2.17	163.46 ± 2.72
4.0	20.24 ± 0.41	101.08 ± 3.39	60.57 ± 1.98	196.81 ± 4.42
5.0	24.98 ± 0.39	124.46 ± 4.76	71.68 ± 2.29	234.77 ± 6.28
6.0	29.07 ± 0.68	145.33 ± 5.98	78.16 ± 2.32	251.92 ± 5.72
7.0	33.84 ± 0.79	161.77 ± 6.98	89.20 ± 4.67	266.54 ± 9.39
8.0	38.49 ± 0.93	179.10 ± 8.56	100.11 ± 1.69	293.75 ± 8.84
9.0	40.67 ± 1.12	189.07 ± 6.93	115.08 ± 5.78	326.34 ± 10.70
10.0	43.63 ± 1.58	208.46 ± 9.93	125.48 ± 2.384	354.80 ± 13.05
11.0	45.26 ± 1.24	223.01 ± 12.80	143.79 ± 5.70	378.48 ± 13.94
12.0	49.78 ± 1.99	247.49 ± 13.65	153.26 ± 3.38	410.72 ± 14.54
24.0	69.78 ± 2.75	345.10 ± 19.12	204.94 ± 6.44	503.84 ± 21.21
**J_ss_**	**4.20**	**18.43**	**6.45**	**35.06**
PER	1	4.38	1.54	8.34

**Table 6:** Pharmacokinetic parameters (FBX) computed using Kinetica Software.

Parameters	Marketed Oral Tablet	Transdermal Film	Cubosomal Gel	MN Patch	Cubosomal Loaded MN Patch
C_max_ (ng/mL)	207.53	97.58	218.44	112.48	271.03
T_max_	2.00	4.00	6.00	5.00	6.00
AUC_0–t_ (ng × h/mL)	2784.06	798.84	3825.48	1957.89	7328.70
T_1/2_ (h)	11.72	6.32	11.28	11.15	18.57
MRT (h)	16.91	9.79	18.43	17.72	29.05
F_rel_	1	0.29	1.37	0.70	2.63

## Data Availability

A data is not available due to privacy reason.

## Supplementary Materials

The following supporting information can be downloaded at: https://www.mdpi.com/article/10.3390/pharmaceutics15010224/s1, Figure S1. Load vs. Time curve for MN patch loaded with cubosomes of FBX; Figure S2. In-vitro dissolution study of MN patch containing FBX loaded cubosomes; Figure S3. Skin penetrability of Cubosomes of FBX loaded MN Patch.
