# Supplementary figures and images for: Formulation Development of Fast Dissolving Microneedles Loaded with Cubosomes of Febuxostat: In Vitro and In Vivo Evaluation
Source: Pharmaceutics. 2023 Jan 9;15(1):224. doi: 10.3390/pharmaceutics15010224 (PMC9863705; doi:10.3390/pharmaceutics15010224)

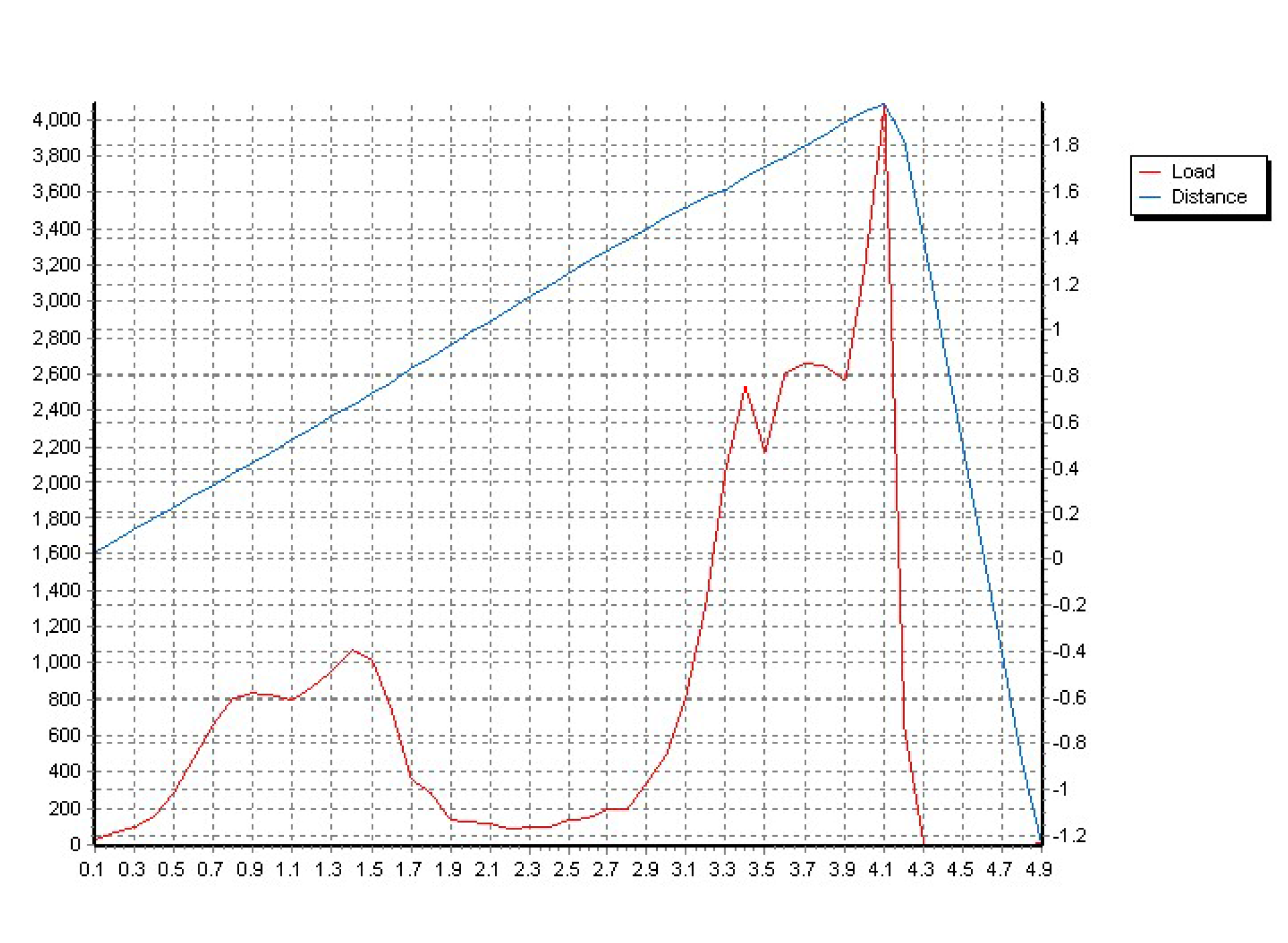

Supplement: Supplementary file 1 [file pharmaceutics-15-00224-s001.zip › Figure S1. Load vs Time curve for MN patch loaded with cubosomes of FBX.tiff]

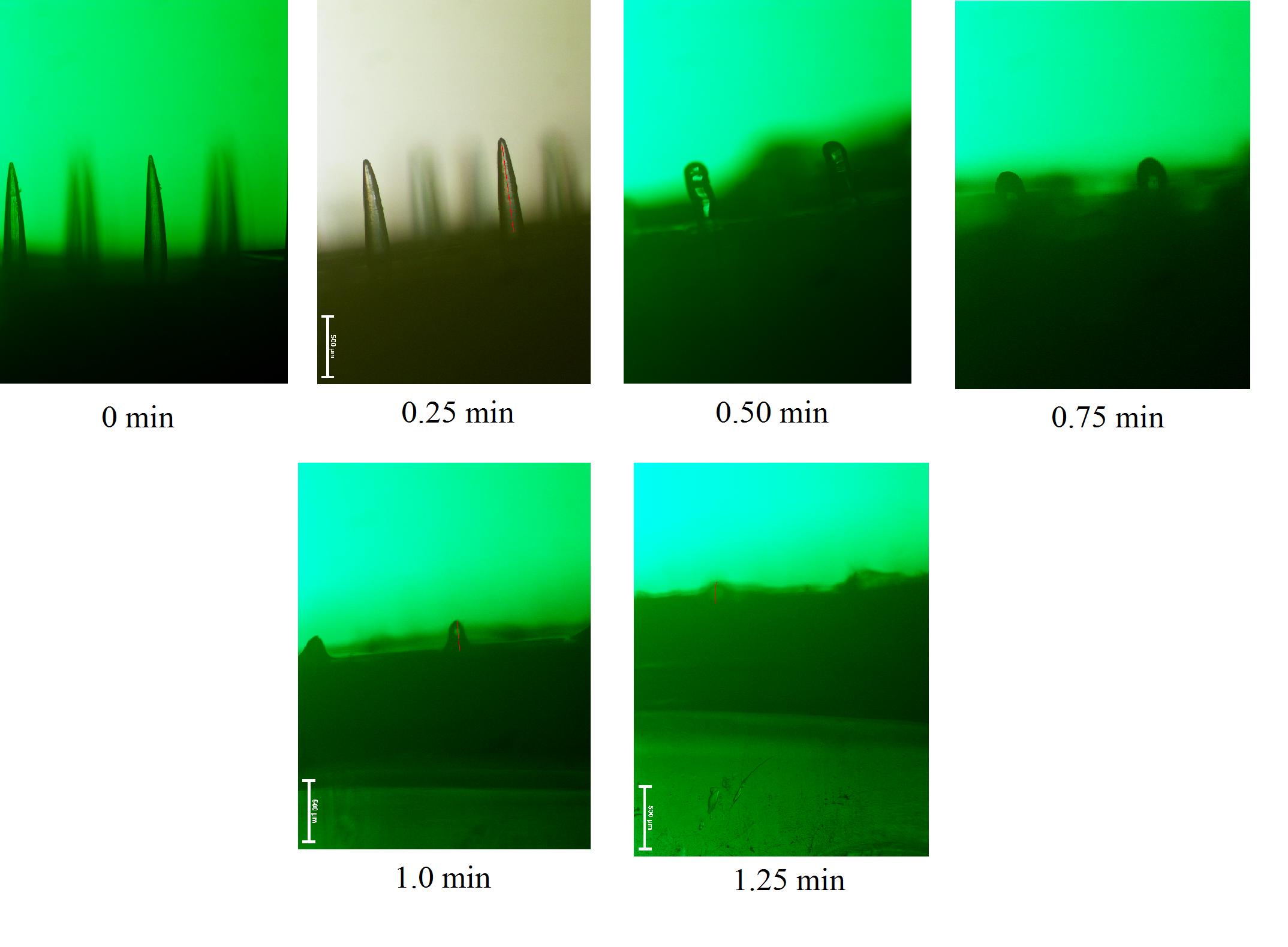

Supplement: Supplementary file 1 [file pharmaceutics-15-00224-s001.zip › Figure S2. In-vitro dissolution study of MN patch containing FBX loaded cubosomes.tif]

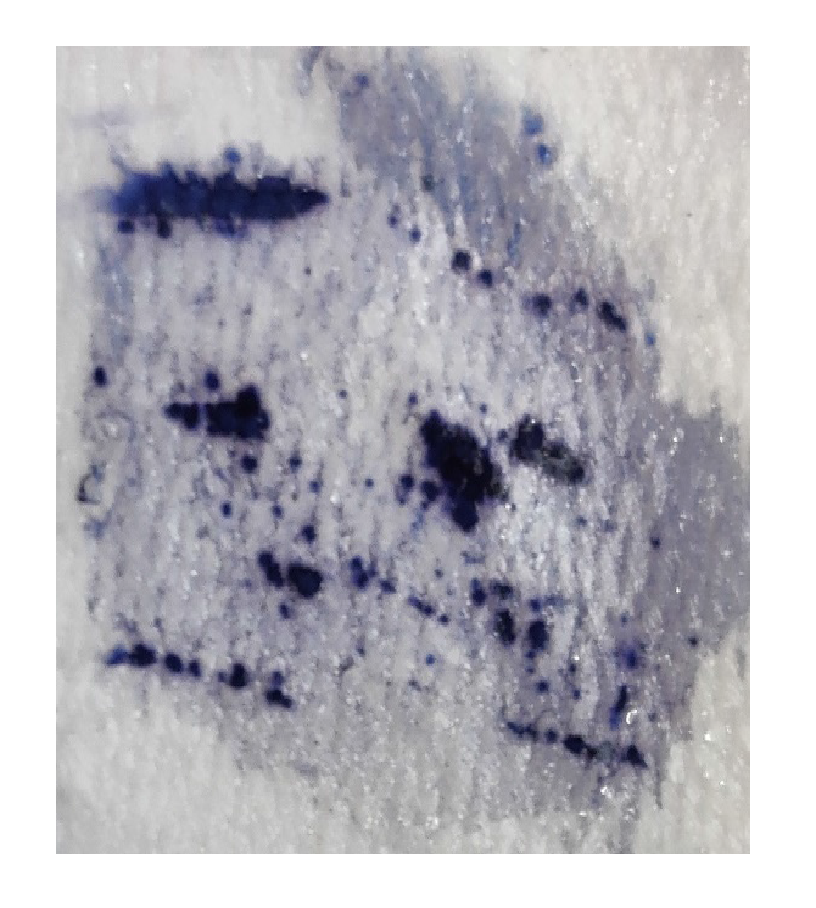

Supplement: Supplementary file 1 [file pharmaceutics-15-00224-s001.zip › Figure S3. Skin penetrability of Cubosomes of FBX loaded MN Patch.tif]
